# Routine *versus* selective intraoperative cholangiography during cholecystectomy: systematic review, meta-analysis and health economic model analysis of iatrogenic bile duct injury

**DOI:** 10.1093/bjsopen/zraa032

**Published:** 2020-12-31

**Authors:** J M L Rystedt, J Wiss, J Adolfsson, L Enochsson, B Hallerbäck, P Johansson, C Jönsson, P Leander, J Österberg, A Montgomery

**Affiliations:** Department of Surgery, Skane University Hospital, Clinical Sciences, Lund University, Sweden; Swedish Agency for Health Technology Assessment and Assessment of Social Services (SBU), Stockholm, Sweden; Swedish Agency for Health Technology Assessment and Assessment of Social Services (SBU), Stockholm, Sweden; Department of Clinical Sciences, Intervention and Technology (CLINTEC), Karolinska Institutet, Stockholm, Sweden; Department of Surgical and Perioperative Sciences, Umeå University, Umeå, Sweden; Department of Surgery, Northern Alvsborg Hospital, Trollhattan, Sweden; PublicHealth&Economics, Stockholm, Sweden; Research Centre for Health and Welfare, Halmstad University, Halmstad, Sweden; Institute of Clinical Sciences, Sahlgrenska Academy, University of Gothenburg, Gothenburg, Sweden; Department of Surgery, Sahlgrenska University Hospital, Gothenburg, Sweden; Department of Radiology, Skane University Hospital, Malmö, Sweden; Department of Surgery, Mora Hospital, Mora, Sweden; Department of Surgery, Skane University Hospital, Clinical Sciences, Lund University, Sweden

## Abstract

**Background:**

Bile duct injury (BDI) is a severe complication following cholecystectomy. Early recognition and treatment of BDI has been shown to reduce costs and improve patients’ quality of life. The aim of this study was to assess the effect and cost-effectiveness of routine *versus* selective intraoperative cholangiography (IOC) in cholecystectomy.

**Methods:**

A systematic review and meta-analysis, combined with a health economic model analysis in the Swedish setting, was performed. Costs per quality-adjusted life-year (QALY) for routine *versus* selective IOC during cholecystectomy for different scenarios were calculated.

**Results:**

In this meta-analysis, eight studies with more than 2 million patients subjected to cholecystectomy and 9000 BDIs were included. The rate of BDI was estimated to 0.36 per cent when IOC was performed routinely, compared with to 0.53 per cent when used selectively, indicating an increased risk for BDI of 43 per cent when IOC was used selectively (odds ratio 1.43, 95 per cent c.i. 1.22 to 1.67). The model analysis estimated that seven injuries were avoided annually by routine IOC in Sweden, a population of 10 million. Over a 10-year period, 33 QALYs would be gained at an approximate net cost of €808 000 , at a cost per QALY of about €24 900.

**Conclusion:**

Routine IOC during cholecystectomy reduces the risk of BDI compared with the selective strategy and is a potentially cost-effective intervention.

## Teaser

This review and meta-analysis confirmed that the use of routine intraoperative cholangiography (IOC) prevents bile duct injury at cholecystectomy. The health economic model showed that routine IOC is a potentially cost-effective intervention compared with the selective strategy of IOC on demand.

Routine intraoperative cholangiography is a potentially cost-effective intervention

## Resumen

**Antecedentes:**

La lesión de la vía biliar (*bile duct injury*, BDI) es una complicación grave de la colecistectomía. Se ha demostrado que la detección y el tratamiento precoz de la BDI reducen los costes y mejoran la calidad de vida del paciente (*quality-of-life*, QoL). El objetivo del estudio fue evaluar el efecto y el coste-beneficio de la colangiografía intraoperatoria (*intraoperative cholangiography*, IOC) de rutina *versus* la IOC selectiva durante la colecistectomía.

**Métodos:**

Se realizó una revisión sistemática y un metaanálisis combinados con un análisis del modelo económico de salud en el entorno sueco. Se calculó el coste por año de vida ajustado por calidad (*quality-adjusted life-year*, QALY) para la IOC de rutina *versus* la IOC selectiva durante la colecistectomía para diferentes escenarios.

**Resultados:**

En este metaanálisis se incluyeron ocho estudios con más de 2 millones de pacientes sometidos a colecistectomía y 9.000 BDIs. La tasa de BDI se estimó en un 0,36% cuando la IOC se realizó de forma rutinaria, en comparación con un 0,53% cuando se utilizó de forma selectiva, lo que implicaba un aumento del riesgo de BDI del 43% cuando se utilizó la IOC de forma selectiva (razón de oportunidades, odds ratio, OR 1,43; i.c. del 95% 1,22 a 1,67). El análisis del modelo económico estimó que la IOC de rutina evitaba siete lesiones al año en Suecia, una población de 10 millones de personas. Durante un periodo de diez años, se obtendrían 33 QALYs a un coste neto de 808.000 € con un coste por QALY de 24.900 €.

**Conclusión:**

La IOC de rutina durante la colecistectomía reduce el riesgo de BDI en comparación con la estrategia de la realización selectiva y es una intervención potencialmente rentable.

## Introduction

Cholecystectomy, performed as an emergency or elective procedure, is one of the most common abdominal operations performed by general surgeons’ worldwide^1^^,^^2^. One of the most feared complications, bile duct injury (BDI), is reported to occur in 0.3–1.5 per cent of procedures^1^^,^^3–5^. A major BDI may result in considerable short- and long-term morbidity, and is sometimes fatal. Roux-en-Y hepaticojejunostomy is the treatment of choice for severe BDI, and a few patients need liver transplantation^1^^,^^3^^,^^6^^,^^7^. Routine intraoperative cholangiography (IOC) has been estimated to prevent 2.5 deaths per 10 000 cholecystectomies, and is reported to increase the number of patients having an intraoperative diagnosis^8–10^. Immediate diagnosis of a BDI will potentially lead to lower costs for the healthcare system and society resulting in better patient-reported quality of life (QoL)^9–12^. Available literature on costs and outcomes after BDI come mostly from referral centres, with a selection of major injuries and failures after primary repair, or theoretical models^8^^,^^11–13^.

In Sweden, IOC is considered a routine procedure, compared with most countries where IOC is performed selectively, on demand. More than 13 000 cholecystectomies are performed annually in Sweden, with a population of 10 million. Treatment of gallstones, surgically and endoscopically, is registered in the Swedish Registry of Gallstone Surgery and Endoscopic Retrograde Cholangiopancreatography (GallRiks) with a national coverage greater than 85 per cent since 2009^14^.

The rationale behind performing IOC routinely in Sweden is for the detection of stones in the common bile duct (CBD) to be dealt with during surgery, as well as having an early diagnosis of iatrogenic injury to the CBD, avoiding more severe injury including substance loss^5^^,^^9^^,^^15^. IOC can also aid in avoiding visual misinterpretation of the anatomy, thereby reducing the risk of an injury, and is used in Sweden for documentation of the anatomy in medical records^15^^,^^16^. Recently, the Swedish Surgical Society and the Swedish Agency for Health Technology Assessment and Assessment of Social Services (SBU) published a systematic review in Swedish (Intraoperative cholangiography in cholecystectomy) that forms the basis of this paper^17^.

The aim of this systematic review and analysis was to evaluate the potential benefits, risks and cost-effectiveness of shifting from routine to selective IOC at cholecystectomy. A health economic model was constructed to calculate the incremental cost-effectiveness for routine *versus* selective IOC.

## Methods

A systematic review of the literature was performed to compare routine and selective strategies for IOC during cholecystectomy. The review process followed the PRISMA statement^18^.

Evidence was evaluated according to the GRADE recommendations (http://www.gradeworkinggroup.org/), and a health economic model analysis was performed. Questions according to the PICO (population, intervention, control, outcome) process were: population, patients subject to cholecystectomy; intervention, IOC; control, selective or no IOC during cholecystectomy; outcome, BDIs, total length of stay, complications from the intervention (IOC).

Principal summary measures for the meta-analysis were the rate of BDI and the odds ratio (OR) for BDI using IOC selectively compared with the routine strategy during cholecystectomy. The meta-analysis was done in RevMan 5.3 (The Cochrane Collaboration, The Nordic Cochrane Centre, Copenhagen, Denmark) and is presented as a forest plot.

### Study selection criteria

Studies published from 1990 were included if they met the following criteria: RCT; controlled trial (effect); population-based observational study; and recently published systematic review. Only studies in English or Scandinavian languages were included. The literature search was performed in PubMed, EMBASE and the Cochrane Library in November and December 2014, and updated in February 2018 (*Appendix S1*).

Screening of abstracts, and assessment of relevance and risk of bias was done independently by two individuals from the expert group or SBU^17^. Data extraction was performed jointly by an expert group (5 surgeons and 1 radiologist) and specialists from SBU. Risk of bias was assessed using the SBU standard for risk of bias in observational studies. Results were discussed jointly in the expert group.

### Quality of life

The EQ-5D™ (EuroQol Group, Rotterdam, the Netherlands) is a standardized instrument for measuring patients’ QoL, used when calculating quality-adjusted life-years (QALYs)^19–22^. QALYs incorporate changes in both quantity (longevity/mortality) and quality (morbidity, psychological, functional, social, and other factors) of life. Perfect health for 1 year in equals 1 QALY and death equals 0 QALY. The different QoL weights used were retrieved from the EQ-5D™ index reported by Rystedt and colleagues^9^, with a median follow-up of 4.3 years. A minor BDI was defined as a lesion smaller than 5 mm, corresponding to Hannover grade C1 and Strasberg type D, including iatrogenic injury when introducing an IOC catheter to the CBD^3^^,^^23^. A major BDI was defined as a lesion greater than 5 mm, total division of the CBD or of one of its major branches, or transection of the central ducts. Leakage from the cystic duct or liver bed (Hannover grade A and Strasberg grade A) was not included^3^^,^^23^.

EQ-5D™ index values used in the model were: minor BDI detected during surgery, 0.9; major BDI detected during surgery, 0.86; minor BDI detected after surgery,0.83; major BDI detected after surgery, 0.67)^9^. A patient with no BDI was assumed to have the same QoL as a patient with a minor BDI detected during surgery (0.9).

### Health economic analysis

**Fig. 1:**
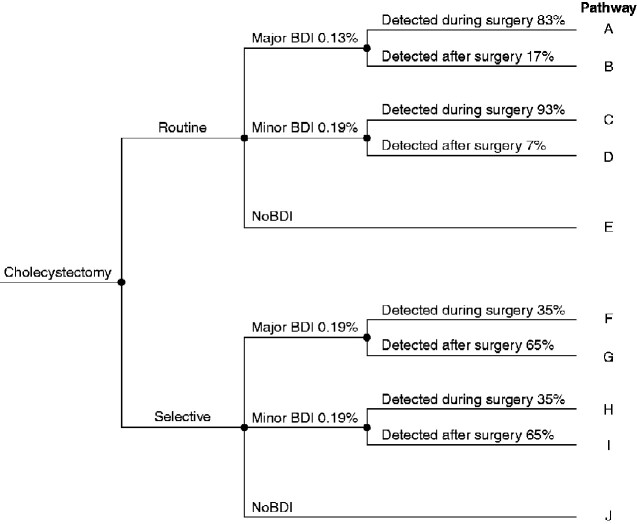
Decision tree for the health economic model of routine and selective intraoperative cholangiography during cholecystectomy with different rates of bile duct injury BDI, bile duct injury.

A deterministic model (decision tree) was constructed to evaluate the cost-effectiveness of routine *versus* selective IOC (*Fig. 1*). The model was adopted to Swedish conditions estimating the risk for a minor or major BDI separately, and whether the BDI was detected during or after surgery. A 10-year perspective was applied in the analysis to capture the effect on patients’ QoL, expressed as QALYs. The frequency and relative risk (RR) for a BDI at cholecystectomy were extracted from this systematic review/meta-analysis and combined with Swedish national data on costs and QoL^9^. These data were supplemented with the following estimations and assumptions for the strategy of selective IOC: the frequency of selective IOC was set at 40 per cent, an estimation based on the prevalence of complicated gallstone disease in the Swedish cohort and the recommended practice for IOC during cholecystectomy for acute cholecystitis^2^^,^^8^^,^^9^^,^^24^; the proportion of major BDI was estimated to increase from 41 to 50 per cent when IOC was used selectively^25–28^; the proportion of major BDIs detected after surgery was estimated to increase from 17 to 65 per cent when selective IOC was applied^29–31^.

The health economic analysis is presented as the incremental cost-effectiveness ratio (ICER) for routine IOC compared with selective IOC (the difference in total costs divided by the difference in effect—in this case, QALYs).

#### Costs

The unit cost for a cholecystectomy used in the calculations was €4500 for both strategies^17^. The cost of an IOC was estimated to differ between strategies according to the additional time needed to gather and assemble the equipment for fluoroscopy when not using it routinely: 25 min for selective use, resulting in a total of €520, *versus* 12 min for routine IOC, resulting in a total of €330^9^.

The costs associated with a BDI were estimated for each strategy respectively, routine or selective IOC at cholecystectomy, using original Swedish data from Rystedt *et al*.^9^ . The total costs used for BDIs were: €11 200 for a minor BDI and €18 700 for a major BDI detected during surgery; €21 400 for a minor BDI and €42 800 for a major BDI detected after surgery. All costs were converted from Swedish krona to euros (year 2019) using purchasing power parity via the CCEMG – EPPI-Centre Cost Converter^32^.

### Sensitivity analysis

Sensitivity analysis were performed for four different scenarios with assumptions on both absolute and relative risks of BDI for the two strategies, routine and selective IOC. The reference scenario was the RR of suffering from a BDI taken from the meta-analysis of this systematic review. One-way analysis were performed for the following variables (ranges in parentheses): the unit cost of selective IOC (€330–670); the frequency of selective IOC performed (20–60 per cent); the proportion of BDIs detected during surgery (20–50 per cent); the proportion of major BDIs (41–80 per cent); and discount rates (0 and 5 per cent). The time frame was limited to 4 years, as QoL weights were based on the study by Rystedt *et* al.^9^.

## Results

**Fig. 2:**
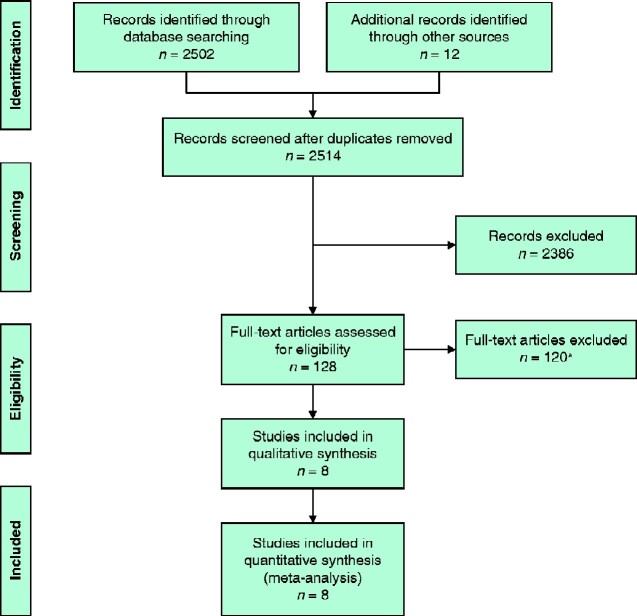
PRISMA diagram for the review *See *Table S1* for details of full-text articles excluded.

Eight observational studies^4^^,^^24^^,^^26^^,^^33–37^ were included in the final analysis (*Fig. 2*). Excluded studies and reasons for exclusion are shown in *Table S1*. Study period, number of patients, primary and secondary outcomes, follow-up, and risk of bias in the included studies are listed in *Table S2*.

**Fig. 3:**
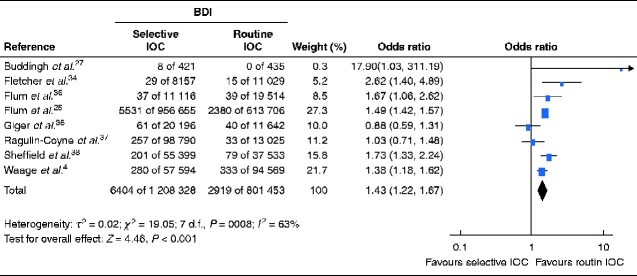
Forest plot of studies comparing bile duct injury after selective *versus* routine intraoperative cholangiography A Mantel–Haenszel random-effects model was used for meta-analysis. Odds ratios are shown with 95 per cent confidence intervals. IOC, intraoperative cholangiography.

For the strategy of routine IOC, the total number of BDIs in the included studies was 2919 of 801 453 cholecystectomies, resulting in a frequency of 0.36 per cent. The selective IOC strategy resulted in 6404 BDIs of 1 208 328 cholecystectomies, giving a frequency of 0.53 per cent. The meta-analysis showed a 43 per cent increased risk of BDI when a selective strategy for IOC was used (OR 1.43, 95 per cent c.i. 1.22 to 1.67) (*Fig. 3*).

### Cost-effectiveness of routine *versus* selective intraoperative cholangiography

Five studies^8^^,^^9^^,^^38–40^ were considered potentially relevant and scrutinized accordingly. Three^9^^,^^39^^,^^40^ of these studies were classified as low quality owing to inadequate methodology or low transferability to Swedish circumstances. However, one study^9^ included detailed data on costs and QoL for BDI with high relevance to the Swedish context, and is why these data were used in this model analysis. In a cohort of 13 156 patients who had a cholecystectomy, routine *versus* selective use of IOC was estimated, over a 10-year period, to lead to seven avoided injuries and 33 QALYs gained in the reference scenario. The ICER was approximately €24 900 for routine IOC compared with selective IOC (*Table 1*).

**Table 1:** Incremental costs, bile duct injuries avoided, and quality-adjusted life-years gained*

	Cost (€)	Difference (routine *versus* selective IOC) (€)
**Cost of IOC**		1 365 541
Routine	4 121 678	
Selective	2 756 137	
**Cost of BDI**		–557 588
Routine	59 545 285	
Selective	60 102 873	
**Total cost**		807 953
Routine	63 666 963	
Selective	62 859 010	
**No. of BDIs avoided**		7
Routine	42	
Selective	49	
**ICER per BDI**	118 558	
**QALYs gained**		33
Routine	102 566	
Selective	102 534	
**ICER per QALY**	24 853	

*The model’s reference scenario had a relative risk for bile duct injury (BDI) of 1.43, in a cohort of 13 156 patients who had a cholecystectomy. Costs for routine *versus* selective intraoperative cholangiography (IOC) in the Swedish setting, over a 10-year period, were compared. ICER, incremental cost-effectiveness ratio; QALY, quality-adjusted life-year.

### Sensitivity analysis

**Fig. 4:**
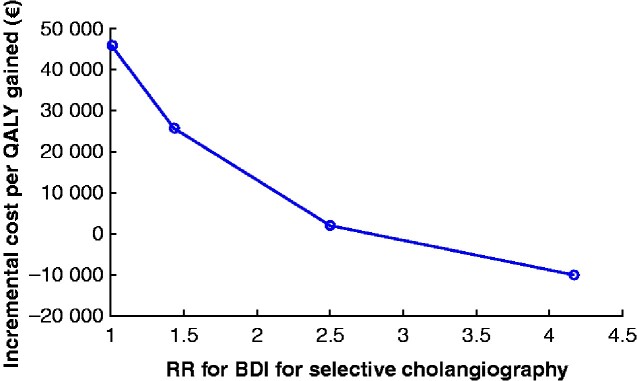
Cost per quality-adjusted life-year varying with the relative risk of bile duct injury QALY, quality-adjusted life-year; RR, relative risk; BDI, bile duct injury.

In the meta-analysis one large study^24^ dominated, but OR did not change when this study was left out in a separate analysis: there was still a 43 per cent increased risk of BDI when a selective strategy for IOC was used (OR 1.43, 95 per cent c.i. 1.11 to 1.83). The incremental cost per QALY gained with routine IOC was very sensitive to the RR for a major BDI (*Fig. 4*). The reference scenario used the RR of 1.43 from this systematic review. However, other possible scenarios were explored based on the BDI rates calculated in the meta-analysis. An alternative scenario used was the absolute risk of BDI for the selective strategy (0.53 per cent) compared to the absolute risk of a major BDI in Sweden today using routine IOC (0.13 per cent), which would be equivalent to a RR for a major BDI of 4.17. If this relative risk of 4.17 was assumed, routine IOC would be the dominant strategy (less costly and more effective). A hypothetical conservative scenario, assuming an equal risk (RR 1.00) of major BDI for both strategies, would result in an incremental cost per QALY close to €45 700.

**Fig. 5:**
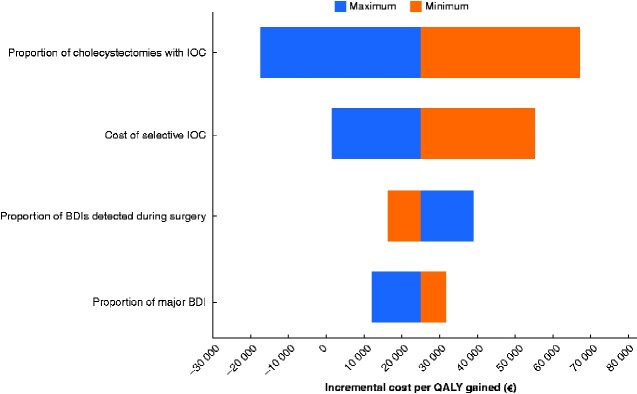
Tornado diagram for sensitivity analysis showing how incremental cost per quality-adjusted life-year varied in the model with changes to the variables IOC, intraoperative cholangiography; BDI, bile duct injury’ QALY, quality-adjusted life-year.

The tornado diagram in *Fig. 5* shows how the incremental cost per QALY varied in the model with changes to the variables. The proportion of cholecystectomies using selective IOC and the ‘unit cost’ for IOC were the factors having the greatest influence on the results. The cost per QALY gained, when using IOC routinely, varied from being a dominant strategy to €67 200 per QALY.

Sensitivity analysis showed little impact when varying the discount rate from 0 to 5 per cent (from €21 900 to €26 900). When the shorter time horizon of 4 years was applied in the calculations (instead of 10 years), QALYs gained decreased to 14 (rather than 33), resulting in an incremental cost per QALY of €56 500.

## Discussion

Despite laparoscopic cholecystectomy being well established, and new imaging modalities and surgical devices being at hand, BDI still remains a clinical problem. The value of routine IOC during cholecystectomy in preventing BDI is disputed. If IOC is continued to be performed routinely during cholecystectomy in Sweden, seven BDIs per year will be avoided according to the results of this study.

This review was originally presented in Swedish in August 2018 to illustrate the current strategy in Sweden of routine IOC and to investigate what the effects of changing to a more selective approach would be^17^. Routine IOC at cholecystectomy has been the standard of care in Sweden for decades, and previous studies from the national quality register GallRiks have shown a high rate of intraoperative detection, with a majority of injuries being minor^5^. However, in most countries the use of routine IOC to prevent BDI remains controversial. When performing IOC, a cut is made into the cystic duct. If the surgeon has misinterpreted the anatomy and cut into the CBD, completing an IOC will hopefully help the team to recognize the error. The immediate diagnosis of a BDI by IOC prevents the surgeon from proceeding and transforming the cut made by scissors into a more severe injury, as a complete transection of the CBD or loss of substance of the extrahepatic biliary tree.

The strength of this study is that the review included more than 2 million patients subjected to cholecystectomy, and that detailed, prospectively collected data from the national Swedish register GallRiks^2^ were used. The lack of data on risk of minor BDI when practising the strategy of selective IOC adds uncertainty to the analysis, and the sensitivity analysis showed a rather large variation. The health economic model has gathered the best available information on costs, QoL, and the risk of having a BDI when using routine *versus* selective IOC. However, the lack of data on several important parameters regarding selective IOC adds uncertainty to the analysis. The sensitivity analysis showed a large variation in the cost per QALY. To give a more precise estimation of the cost per QALY, more (and better quality) data are needed on how a selective approach affects the risk of both minor and major BDI,, costs, and QoL.

Early diagnosis and prompt treatment of BDI has been shown to reduce costs and improve patients’ QoL^9^^,^^41^. More than 20 years ago, Savader and colleagues^12^ described significantly reduced costs (43–83 per cent) for BDIs detected during surgery. For these reasons, many have advocated IOC in high-risk procedures, complicated pathology, or operations by less experienced surgeons to prevent BDI (the selective strategy)^8^. Rates of intraoperative detection of BDI vary greatly, from 20 to 30 per cent in centres practising the selective strategy to 90 per cent reported from centres performing routine IOC^5^^,^^10^^,^^30^^,^^42^^,^^43^. This difference is large. However, in the present sensitivity analysis, the proportion of BDIs detected during surgery had only a moderate effect on the cost per QALY gained.

Postoperative diagnosis of BDI and prolonged treatment have been reported to be factors associated with reduced QoL^41^^,^^44^. Previous publications have favoured early repair by a hepatobiliary surgeon in terms of both clinical outcome and QoL, and cost-effectiveness analysis have described this as the superior strategy^8^^,^^12^^,^^13^^,^^41^^,^^44^. In the present study, the use of routine IOC was found to be a potentially cost-effective intervention by reducing the number of BDIs and increasing the proportion of injuries detected at operation.

An intervention is often considered cost-effective if the ICER is less than the willingness-to-pay (WTP) threshold. The WTP is the maximum amount of money society is willing to spend for 1 QALY (1 year in perfect health), is not set explicitly, and varies between countries and contexts from €25 000 to €80 000^8^^,^^45–48^.

In this study the cost of IOC had a large effect on total costs in the sensitivity analysis, as did the time perspective, using a 4- or 10-year horizon. The consequence of different ‘unit costs for IOC’ is in line with that found previous publications. Flum and co-workers reported the unit cost for IOC to vary in the literature between €90 and €840 (in the year 2019)^8^^,^^32^. Any cost savings secondary to intraoperative detection of CBD stones or reduced rates of cystic duct leakage were not included in this analysis^15^^,^^49^.

This review and meta-analysis has confirmed that the use of routine IOC reduces the risk and prevents BDI at cholecystectomy. The health economic model shows that routine IOC is a potentially cost-effective intervention compared with the selective strategy of IOC on demand.

## Funding

Swedish government

## Supplementary Material

zraa032_Supplementary_Data

## Notes

Presented in part to Swedish Surgical Week, Helsingborg, Sweden, August 2018; published online in Swedish at https://www.sbu.se/292
