# Supplementary material for: Routine versus selective intraoperative cholangiography during cholecystectomy: systematic review, meta-analysis and health economic model analysis of iatrogenic bile duct injury
Source: BJS Open. 2020 Dec 31;5(2):zraa032. doi: 10.1093/bjsopen/zraa032 (PMC7944855; doi:10.1093/bjsopen/zraa032)
Supplement: zraa032_Supplementary_Data [file zraa032_supplementary_data.zip › Supplement 1 Literature search.docx]

# Supplement 1

# Literature Search

### PubMed via NLM 16 February 2018

**Title: Intraoperative cholangiography**

**Search terms**

**Items**

**found**

**Population: persons in surgery for cholecystectomy**

1. Cholecystectomy[MeSH] OR Gallstones/surgery[MeSH] OR Gallbladder/surgery[MeSH] 30464
2. cholecystectomy[tiab] OR cholecystitis[tiab] 35377

3. *1 OR 2 46797*

### Intervention: peroperative

1. "Intraoperative Care/methods"[MeSH Terms] OR "Perioperative Care"[Mesh:NoExp] OR Intraoperative Complications/surgery[MeSH]
2. Peroperative[tiab] OR operative[tiab] OR intraoperative[tiab] OR during[ti] OR intra- operativ*[tiab] OR per-operativ*[tiab] OR cholangiography during surgery[tiab] OR cholangiography during operation[tiab] OR cholangiography during laparoscopic cholecystectomy[tiab]

17931

338909

6. *4 OR 5 351087*

### Intervention: cholangiography

| 7. "Cholangiography"[Mesh:NoExp] | 11694 |
| --- | --- |
| 8. cholangiograp*[tiab] OR cholangiogram*[tiab] OR cholangiographer*[tiab] OR fluorocholangiography[tiab] | 10688 |
| 9. *7 OR 8* | *16402* |
| **Combined sets** |  |
| **10. 3 AND 6 AND 9** | **2826** |

The search result, usually found at the end of the documentation, forms the list of abstracts.

[MeSH] = Term from the Medline controlled vocabulary, including terms found below this term in the MeSH hierarchy [MeSH:NoExp] = Does not include terms found below this term in the MeSH hierarchy

[MAJR] = MeSH Major Topic [TIAB] = Title or abstract [TI] = Title

[AU] = Author [OT]= Other term [TW] = Text Word

Systematic[SB] = Filter for retrieving systematic reviews

* = Truncation

### Embase via Elsevier 16 February 2018

**Title: Intraoperative cholangiography**

**Search terms**

**Items**

**found**

**Population: persons in surgery for cholecystectomy**

1. 'cholecystectomy'/de OR 'gallstone'/dm_su 45,720
2. cholecystectomy:ti,ab OR cholecystitis:ti,ab 44,980

3. *1 OR 2 61,623*

### Intervention: peroperative cholangiography

| 4. 'peroperative cholangiography'/de | 1,839 |
| --- | --- |
| 5. (peroperative:ti OR operative:ti OR intraoperative:ti OR during:ti OR 'intra operative':ti OR | 1,357 |
| 'per operative':ti) AND (cholangiograp*:ti OR cholangiogram*:ti OR cholangiographer*:ti OR |  |
| fluorocholangiography:ti) OR ((cholangiography NEXT/1 surgery):ti) OR ((cholangiography |  |
| NEXT/1 operation):ti) OR ((cholangiography NEXT/2 cholecystectomy):ti) |  |
| 6. *4 OR 5* | *2,749* |
| **Combined sets** |  |
| 7. *3 AND 6* | *1,508* |
| **8.** **7 AND ([danish]/lim OR [english]/lim OR [norwegian]/lim OR [swedish]/lim) AND [1990-2018]/py** | **1,174** |

The search result, usually found at the end of the documentation, forms the list of abstracts.

/de= Term from the EMTREE controlled vocabulary

/exp= Includes terms found below this term in the EMTREE hierarchy

/mj = Major Topic

:ab = Abstract

:au = Author

:ti = Article Title

:ti:ab = Title or abstract

* = Truncation

“ “ = Citation Marks; searches for an exact phrase

### Cochrane Library via Wiley 16 February 2018 (CDSR, DARE & CENTRAL)

**Title: Intraoperative cholangiography**

**Search terms**

**Items**

**found**

**Population: persons in surgery for cholecystectomy**

1. MeSH descriptor: [Cholecystitis] explode all trees OR

(MeSH descriptor: [Gallbladder Diseases] explode all trees AND MeSH descriptor: [Colic] explode all trees)

OR

MeSH descriptor: [Gallstones] explode all trees]

547

1. (cholecystectomy OR cholecystitis):ti 2942

3. *1 OR 2 3236*

### Intervention: peroperative

1. MeSH descriptor: [Intraoperative Care] explode all trees and with qualifier(s): [Methods - MT]

OR

MeSH descriptor: [Perioperative Care] this term only OR

MeSH descriptor: [Intraoperative Complications] explode all trees

1. (peroperative OR operative OR intraoperative OR during OR intra-operativ* OR per- operativ* OR "cholangiography during surgery" OR "cholangiography during operation" OR "cholangiography during laparoscopic cholecystectomy"):ti, ab, kw

5661

272655

6. *4 OR 5 273840*

### Intervention: cholangiography

1. MeSH descriptor: [Cholangiography] this term only 150
2. (cholangiograp* OR cholangiogram* OR cholangiographer* OR fluorocholangiography):ti, ab, kw

427

9. 7 OR 8 427

[Combined sets](#_TOC_250000)

10. 3 AND 6 AND 9 105

### 11. 10 AND Publication Year from 1990 to 2018 CDSR/0 DARE/3 Central/ 85 CRM/0 HTA/0 EED/8

The search result, usually found at the end of the documentation, forms the list of abstracts. [AU] = Author

[MAJR] = MeSH Major Topic

[MeSH] = Term from the Medline controlled vocabulary, including terms found below this term in the MeSH hierarchy [MeSH:NoExp] = Does not include terms found below this term in the MeSH hierarchy

Systematic[SB] = Filter for retrieving systematic reviews [TI] = Title

[TIAB] = Title or abstract [TW] = Text Word

* = Truncation

“ “ = Citation Marks; searches for an exact phrase

CDSR = Cochrane Database of Systematic Review

CENTRAL = Cochrane Central Register of Controlled Trials, “trials” CRM = Method Studies

DARE = Database Abstracts of Reviews of Effects, “other reviews” EED = Economic Evaluations

HTA = Health Technology Assessments
