# Supplementary material for: Routine versus selective intraoperative cholangiography during cholecystectomy: systematic review, meta-analysis and health economic model analysis of iatrogenic bile duct injury
Source: BJS Open. 2020 Dec 31;5(2):zraa032. doi: 10.1093/bjsopen/zraa032 (PMC7944855; doi:10.1093/bjsopen/zraa032)
Supplement: zraa032_Supplementary_Data [file zraa032_supplementary_data.zip › Supplement 2 Excluded studies.docx]

**Supplement 2** Excluded studies and reason for exclusion

|  |  |  |
| --- | --- | --- |
| **Article** | | **Reason for exclusion** |
| Adams DB,Borowicz MR,Wootton FT Cunningham JT. Bile duct complications after laparoscopic cholecystectomy. Surg Endosc, 1993; 779-83. | | PICO |
| Airan M,Arregui M,Berci G,Cuschieri A,Hunter J,Ko ST, et al. Routine operative cholangiography in patients undergoing laparoscopic cholecystectomy. Ann Surg, 1993; 218216-8. | | PICO |
| Akolekar D,Nixon SJ, Parks RW. Intraoperative cholangiography in modern surgical practice. Dig Surg, 2009; 26130-4. | | PICO, study design |
| Alinder G,Nilsson U,Lunderquist A,Herlin P, Holmin T. Pre-operative infusion cholangiography compared to routine operative cholangiography at elective cholecystectomy. Br J Surg, 1986; 73383-7. | | Time period |
| Al-Qasabi Q,Mofti AB,Suleiman SI,Al-Momen A, Anwar IM. Operative cholangiography in laparoscopic cholecystectomy: Is it essential? Ann Saudi Med, 1997; 17167-9. | | PICO, study design |
| Alvarez FA,de S,Palavecino M,Sanchez C,Mazza O,Arbues G, et al. Impact of routine intraoperative cholangiography during laparoscopic cholecystectomy on bile duct injury. Br J Surg, 2014; 101677-84. | | PICO, study design |
| Ammori MB, Al-Dabbagh AK. Laparoscopic cholecystectomy without intraoperative cholangiography. J Laparoendosc Adv Surg Tech A, 2012; 22146-51. | | Study design |
| Amott D,Webb A, Tulloh B. Prospective comparison of routine and selective operative cholangiography. ANZ J Surg, 2005; 75378-82. | | PIC |
| Ausania F,Holmes LR,Ausania F,Iype S,Ricci P, White SA. Intraoperative cholangiography in the laparoscopic cholecystectomy era: why are we still debating? Surg Endosc, 2012; 261193-200. | | Study design |
| Biffl WL,Moore EE,Offner PJ,Franciose RJ, Burch JM. Routine intraoperative laparoscopic ultrasonography with selective cholangiography reduces bile duct complications during laparoscopic cholecystectomy. J Am Coll Surg, 2001; 193272-80. | | PICO, study design |
| Bilimoria KY,Chung J, Soper NJ. Laparoscopic cholecystectomy, intraoperative cholangiograms, and common duct injuries. Jama, 2013; 310801-2. | | PICO, study design |
| Birth M,Ehlers KU,Delinikolas K, Weiser HF. Prospective randomized comparison of laparoscopic ultrasonography using a flexible-tip ultrasound probe and intraoperative dynamic cholangiography during laparoscopic cholecystectomy. Surg Endosc, 1998; 1230-6. | | PICO |
| Borjeson J,Liu SK,Jones S, Matolo NM. Selective intraoperative cholangiography during laparoscopic cholecystectomy: how selective? Am Surg, 2000; 66616-8. | | PICO, study design |
| Braghetto I,Debandi A,Korn O, Bastias J. Long-term follow-up after laparoscopic cholecystectomy without routine intraoperative cholangiography. Surg Laparosc Endosc, 1998; 8349-52. | | PICO, study design |
| Bresadola V,Intini S,Terrosu G,Baccarani U,Marcellino MG,Sistu M, et al. Intraoperative cholangiography in laparoscopic cholecystectomy during residency in general surgery. Surg Endosc, 2001; 15812-5. | | PICO, study design |
| Buanes T,Waage A,Mjaland O, Solheim K. Bile leak after cholecystectomy significance and treatment: results from the National Norwegian Cholecystectomy Registry. Int Surg, 1996; 81276-9. | | PICO |
| Buddingh KT,Morks AN,ten Cate H,H O,Blaauw CB,van D, et al. Documenting correct assessment of biliary anatomy during laparoscopic cholecystectomy. Surg Endosc, 2012; 2679-85. | | PICO, study design |
| Buddingh KT,Nieuwenhuijs VB,van B,Hulscher JB,de J,J S, et al. Intraoperative assessment of biliary anatomy for prevention of bile duct injury: a review of current and future patient safety interventions. Surg Endosc, 2011; 252449-61. | | Study design |
| Caratozzolo E,Massani M,Recordare A,Bonariol L,Antoniutti M,Jelmoni A, et al. Usefulness of both operative cholangiography and conversion to decrease major bile duct injuries during laparoscopic cholecystectomy. J Hepatobiliary Pancreat Surg, 2004; 11171-5. | | PICO, study design |
| Carbonell AM,Lincourt AE,Kercher KW,Matthews BD,Cobb WS,Sing RF, et al. Do patient or hospital demographics predict cholecystectomy outcomes? A nationwide study of 93,578 patients. Surg Endosc, 2005; 19767-73. | | High risk of bias |
| Carroll BJ,Friedman RL,Liberman MA, Phillips EH. Routine cholangiography reduces sequelae of common bile duct injuries. Surg Endosc, 1996; 101194-7. | | High risk of bias |
| Cates JA,Tompkins RK,Zinner MJ,Busuttil RW,Kallman C, Roslyn JJ. Biliary complications of laparoscopic cholecystectomy. Am Surg, 1993; 59243-7. | | PICO, study design |
| Catheline J,Rizk N, Champault G. A comparison of laparoscopic ultrasound versus cholangiography in the evaluation of the biliary tree during laparoscopic cholecystectomy. Eur J Ultrasound, 1999; 101-9. | | PICO, study design |
| Charfare H, Cheslyn-Curtis S. Selective cholangiography in 600 patients undergoing cholecystectomy with 5-year follow-up for residual bile duct stones. Ann R Coll Surg Engl, 2003; 85167-73. | | PICO, study design |
| Chattopadhyay TK,Gupta S,Kumar A, Kapoor VK. Peroperative cholangiogram--routine or selective results of a prospective study. Trop Gastroenterol, 1992; 1375-7. | | Study design |
| Clair DG,Carr-Locke DL,Becker JM, Brooks DC. Routine cholangiography is not warranted during laparoscopic cholecystectomy. Arch Surg, 1993; 128551-4; discussion 54. | | Study design |
| Cockbain AJ,Young AL, Toogood GJ. Randomized clinical trial of routine on-table cholangiography during laparoscopic cholecystectomy (Br J Surg 2011; 98: 362-367). Br J Surg, 2011; 98744; author reply 44. | | PICO, study design |
| Cohen RV,Schiavon CA, Schaffa TD. Is laparoscopic cholecystectomy without intraoperative cholangiography a safe operation? Surg Laparosc Endosc, 1995; 5165-6. | | PICO, study design |
| Connor S, Garden OJ. Bile duct injury in the era of laparoscopic cholecystectomy. Br J Surg, 2006; 93158-68. | | Study design |
| Corbitt JD,Jr, Leonetti LA. One thousand and six consecutive laparoscopic intraoperative cholangiograms. Jsls, 1997; 113-6. | | PICO, study design |
| Csendes A,Burdiles P,Diaz JC,Maluenda F,Korn O,Vallejo E, et al. Prevalence of common bile duct stones according to the increasing number of risk factors present. A prospective study employing routinely intraoperative cholangiography in 477 cases. Hepatogastroenterology, 1998; 451415-21. | | PICO, study design |
| Debru E,Dawson A,Leibman S,Richardson M,Glen L,Hollinshead J, et al. Does routine intraoperative cholangiography prevent bile duct transection? Surg Endosc, 2005; 19589-93. | | Study design |
| Detry O,De R,Detroz B, Honore P. The role of intraoperative cholangiography in detecting and preventing bile duct injury during laparoscopic cholecystectomy. Acta Chir Belg, 2003; 103161-2. | | PICO, study design |
| Enochsson L,Thulin A,Osterberg J,Sandblom G, Persson G. The Swedish Registry of Gallstone Surgery and Endoscopic Retrograde Cholangiopancreatography (GallRiks): A nationwide registry for quality assurance of gallstone surgery. JAMA Surg, 2013; 148471-8. | | Study design |
| Fiore NF,Ledniczky G,Wiebke EA,Broadie TA,Pruitt AL,Goulet RJ, et al. An analysis of perioperative cholangiography in one thousand laparoscopic cholecystectomies. Surgery, 1997; 122817-21; discussion 21. | | Study design |
| Fligelstone L,Wanendeya N, Palmer B. Value of routine intraoperative cholangiography in detecting aberrant bile ducts and bile duct injury during laparoscopic cholecystectomy. Br J Surg, 1996; 831014. | | PICO, study design |
| Fogli L,Boschi S,Patrizi P,Berta RD,Al S,Capizzi D, et al. Laparoscopic cholecystectomy without intraoperative cholangiography: audit of long-term results. J Laparoendosc Adv Surg Tech A, 2009; 19191-3. | | Study design |
| Ford JA,Soop M,Du J,Loveday BP, Rodgers M. Systematic review of intraoperative cholangiography in cholecystectomy. Br J Surg, 2012; 99160-7. | | PICO, study design |
| Haglund U. [Bile duct injury is a disaster for both the patient and the surgeon. Routine intraoperative radiography in cholecystectomy is recommended]. Lakartidningen, 2001; 985620-2. | | PICO, study design |
| Haglund U, Noren A. Routine intraoperative cholangiography in elective laparoscopic cholecystectomy. Scand J Surg, 2010; 99195-6. | | PICO, study design |
| Hamad MA,Nada AA,Abdel-Atty MY, Kawashti AS. Major biliary complications in 2,714 cases of laparoscopic cholecystectomy without intraoperative cholangiography: a multicenter retrospective study. Surg Endosc, 2011; 253747-51. | | PICO, study design |
| Hamouda AH,Goh W,Mahmud S,Khan M, Nassar AH. Intraoperative cholangiography facilitates simple transcystic clearance of ductal stones in units without expertise for laparoscopic bile duct surgery. Surg Endosc, 2007; 21955-9. | | PICO, study design |
| Hauer-Jensen M,Karesen R,Nygaard K,Solheim K,Amlie E,Havig O, et al. Consequences of routine peroperative cholangiography during cholecystectomy for gallstone disease: a prospective, randomized study. World J Surg, 1986; 10996-1002. | | Time period |
| Hauer-Jensen M,Karesen R,Nygaard K,Solheim K,Amlie EJ,Havig O, et al. Prospective randomized study of routine intraoperative cholangiography during open cholecystectomy: long-term follow-up and multivariate analysis of predictors of choledocholithiasis. Surgery, 1993; 113318-23. | | Time period |
| Hobbs MS,Mai Q,Knuiman MW,Fletcher DR, Ridout SC. Surgeon experience and trends in intraoperative complications in laparoscopic cholecystectomy. Br J Surg, 2006; 93844-53. | | Study design |
| Hookman P,Unger SW, Barkin JS. Laparoscopic cholecystectomy should be routinely performed with intraoperative cholangiography. Am J Gastroenterol, 2000; 953299-302. | | PICO, study design |
| Horwood J,Akbar F,Davis K, Morgan R. Prospective evaluation of a selective approach to cholangiography for suspected common bile duct stones. Ann R Coll Surg Engl, 2010; 92206-10. | | Study design |
| Huesch MD, Romley J. Intraoperative cholangiography during cholecystectomy. Jama, 2013; 3102672-4. | | PICO, study design |
| Huguier M,Bornet P,Charpak Y,Houry S, Chastang C. Selective contraindications based on multivariate analysis for operative cholangiography in biliary lithiasis. Surg Gynecol Obstet, 1991; 172470-4. | | Study design |
| Jorgensen JO,Norman SL, Hunt DR. A prospective audit of selective cholangiography for laparoscopic cholecystectomy. Aust N Z J Surg, 1996; 66441-4. | | Study design |
| Khalili TM,Phillips EH,Berci G,Carroll BJ,Gabbay J, Hiatt JR. Final score in laparoscopic cholecystectomy. Cholangiogram 1207, no cholangiogram 116. Surg Endosc, 1997; 111095-8. | | PICO, study design |
| Khan OA,Balaji S,Branagan G,Bennett DH, Davies N. Randomized clinical trial of routine on-table cholangiography during laparoscopic cholecystectomy. Br J Surg, 2011; 98362-7. | | PICO, study design |
| Kohn A,Creech S, Shayani V. Indicated cholangiography in patients operated on by routine versus selective cholangiographers. Am Surg, 2004; 70203-6; discussion 06. | | Study design |
| Kullman E,Borch K,Lindstrom E,Svanvik J, Anderberg B. Value of routine intraoperative cholangiography in detecting aberrant bile ducts and bile duct injuries during laparoscopic cholecystectomy. Br J Surg, 1996; 83171-5. | | PICO, study design |
| Kullman E,Borch K,Lindstrom E,Svanvik J, Anderberg B. Management of bile duct stones in the era of laparoscopic cholecystectomy: appraisal of routine operative cholangiography and endoscopic treatment. Eur J Surg, 1996; 162873-80. | | PICO, study design |
| Ladocsi LT,Benitez LD,Filippone DR, Nance FC. Intraoperative cholangiography in laparoscopic cholecystectomy: a review of 734 consecutive cases. Am Surg, 1997; 63150-6. | | Study design |
| Larsson M, Raf L. [Peroperative cholangiography is a good weapon against injuries. Surgery of gallstones is still done without access to radiography]. Lakartidningen, 1996; 932165-6. | | PICO, study design |
| Larsson M, Raf L. [High number of bile duct injuries in cholecystectomy. There is a connection with the use of the laparoscopic technique]. Lakartidningen, 2001; 985639-42. | | PICO, study design |
| Ledniczky G,Fiore N,Bognar G,Ondrejka P, Grosfeld JL. Evaluation of perioperative cholangiography in one thousand laparoscopic cholecystectomies. Chirurgia (Bucur), 2006; 101 (3): 267-72. | | PICO, study design |
| Lepner U, Grunthal V. Intraoperative cholangiography can be safely omitted during laparoscopic cholecystectomy: a prospective study of 413 consecutive patients. Scand J Surg, 2005; 94197-200. | | PICO, study design |
| Lezoche E,Paganini A, Carle F. Routine versus selective intra-operative cholangiography during laparoscopic cholecystectomy. World J Surg, 1993; 17686-7. | | PICO, study design |
| Lezoche E,Paganini A,Guerrieri M,Carlei F,Lomanto D,Sottili M, et al. Technique and results of routine dynamic cholangiography during 528 consecutive laparoscopic cholecystectomies. Surg Endosc, 1994; 81443-7. | | PICO, study design |
| Lill S,Rantala A,Pekkala E,Sarparanta H,Huhtinen H,Rautava P, et al. Elective laparoscopic cholecystectomy without routine intraoperative cholangiography: a retrospective analysis of 1101 consecutive cases. Scand J Surg, 2010; 99197-200. | | High risk of bias |
| Livingston EH,Miller JA,Coan B, Rege RV. Indications for selective intraoperative cholangiography. J Gastrointest Surg, 2005; 91371-7. | | Study design |
| Livingston EH,Miller JA,Coan B, Rege RV. Costs and utilization of intraoperative cholangiography. J Gastrointest Surg, 2007; 111162-7. | | PICO, study design |
| Ludwig K,Bernhardt J, Lorenz D. Value and consequences of routine intraoperative cholangiography during cholecystectomy. Surg Laparosc Endosc Percutan Tech, 2002; 12154-9. | | High risk of bias |
| Ludwig K,Bernhardt J,Steffen H, Lorenz D. Contribution of intraoperative cholangiography to incidence and outcome of common bile duct injuries during laparoscopic cholecystectomy. Surg Endosc, 2002; 161098-104. | | Study design |
| Manson JM. Intraoperative cholangiography and bile duct injury in laparoscopic cholecystectomy. Surg Endosc, 2000; 1494-5. | | PICO, study design |
| Massarweh NN,Devlin A,Elrod JA,Symons RG, Flum DR. Surgeon knowledge, behavior, and opinions regarding intraoperative cholangiography. J Am Coll Surg, 2008; 207821-30. | | PICO, study design |
| Massarweh NN, Flum DR. Role of intraoperative cholangiography in avoiding bile duct injury. J Am Coll Surg, 2007; 204656-64. | | Study design |
| McFarlane ME,Thomas CA,McCartney T,Bhoorasingh P,Smith G,Lodenquai P, et al. Selective operative cholangiography in the performance of laparoscopic cholecystectomy. Int J Clin Pract, 2005; 591301-3. | | Study design |
| Metcalfe MS,Ong T,Bruening MH,Iswariah H,Wemyss-Holden SA, Maddern GJ. Is laparoscopic intraoperative cholangiogram a matter of routine? Am J Surg, 2004; 187475-81. | | Study design |
| Millat B,Deleuze A,de S,de S, Fingerhut A. Routine intraoperative cholangiography is feasible and efficient during laparoscopic cholecystectomy. Hepatogastroenterology, 1997; 4422-7. | | PICO, study design |
| Mir IS,Mohsin M,Kirmani O,Majid T,Wani K,Hassan MU, et al. Is intra-operative cholangiography necessary during laparoscopic cholecystectomy? A multicentre rural experience from a developing world country. World J Gastroenterol, 2007; 134493-7. | | PICO, study design |
| Mohandas S, John AK. Role of intra operative cholangiogram in current day practice. Int J Surg, 2010; 8602-5. | | Study design |
| Murison MS,Gartell PC, McGinn FP. Does selective peroperative cholangiography result in missed common bile duct stones? J R Coll Surg Edinb, 1993; 38220-4. | | High risk of bias |
| Nickkholgh A,Soltaniyekta S, Kalbasi H. Routine versus selective intraoperative cholangiography during laparoscopic cholecystectomy: a survey of 2,130 patients undergoing laparoscopic cholecystectomy. Surg Endosc, 2006; 20868-74. | | High risk of bias |
| Nies C,Bauknecht F,Groth C,Clerici T,Bartsch D,Lange J, et al. [Intraoperative cholangiography as a routine method? A prospective, controlled, randomized study]. Der Chirurg; Zeitschrift für alle Gebiete der operativen Medizen, 1997; 68892-7. | | High risk of bias |
| Nieuwenhuijs VB. Impact of routine intraoperative cholangiography during laparoscopic cholecystectomy on bile duct injury (Br J Surg 2014; 101: 677-684). Br J Surg, 2014; 101685. | | PICO, study design |
| Nuzzo G,Giuliante F,Giovannini I,Ardito F,D'Acapito F,Vellone M, et al. Bile duct injury during laparoscopic cholecystectomy: results of an Italian national survey on 56 591 cholecystectomies. Arch Surg, 2005; 140986-92. | | High risk of bias |
| Olsen D. Bile duct injuries during laparoscopic cholecystectomy. Surg Endosc, 1997; 11133-8. | | PICO, study design |
| Pesce A,Portale TR,Minutolo V,Scilletta R,Li D, Puleo S. Bile duct injury during laparoscopic cholecystectomy without intraoperative cholangiography: a retrospective study on 1,100 selected patients. Dig Surg, 2012; 29310-4. | | PICO, study design |
| Pickuth D. Selected versus routine use of intraoperative cholangiography during laparoscopic cholecystectomy. Z Gastroenterol, 1995; 33701-3. | | Study design |
| Podnos YD,Gelfand DV,Dulkanchainun TS,Wilson SE,Cao S,Ji P, et al. Is intraoperative cholangiography during laparoscopic cholecystectomy cost effective? Am J Surg, 2001; 182663-9. | | Study design |
| Polat FR,Abci I,Coskun I, Uranues S. The importance of intraoperative cholangiography during laparoscopic cholecystectomy. Jsls, 2000; 4103-7. | | PICO, study design |
| Regoly-Merei J,Ihasz M,Szeberin Z,Sandor J, Mate M. Biliary tract complications in laparoscopic cholecystectomy. A multicenter study of 148 biliary tract injuries in 26,440 operations. Surg Endosc, 1998; 12294-300. | | PICO, study design |
| Robinson BL,Donohue JH,Gunes S,Thompson GB,Grant CS,Sarr MG, et al. Selective operative cholangiography. Appropriate management for laparoscopic cholecystectomy. Arch Surg, 1995; 130625-30; discussion 30. | | Study design |
| Rosen MJ, Ponsky JL. Should intraoperative cholangiography be routinely attempted during laparoscopic cholecystectomy? Nat Clin Pract Gastroenterol Hepatol, 2007; 416-7. | | Study design |
| Rosenthal RJ,Steigerwald SD,Imig R, Bockhorn H. Role of intraoperative cholangiography during endoscopic cholecystectomy. Surg Laparosc Endosc, 1994; 4171-4. | | High risk of bias |
| Sajid MS,Leaver C,Haider Z,Worthington T,Karanjia N, Singh KK. Routine on-table cholangiography during cholecystectomy: a systematic review. Ann R Coll Surg Engl, 2012; 94375-80. | | Study design |
| Sanjay P,Fulke JL, Exon DJ. 'Critical view of safety' as an alternative to routine intraoperative cholangiography during laparoscopic cholecystectomy for acute biliary pathology. J Gastrointest Surg, 2010; 141280-4. | | PICO, study design |
| Shah JN, Shah C. A five years review intra-operative cholangiogram. J Nepal Health Res Counc, 2011; 952-5. | | PICO, study design |
| Sharma AK,Cherry R, Fielding JW. A randomised trial of selective or routine on-table cholangiography. Ann R Coll Surg Engl, 1993; 75245-8. | | PICO, study design |
| Sharma J, Lowenfels AB. Letter 1: Randomized clinical trial of routine on-table cholangiography during laparoscopic cholecystectomy (Br J Surg 2011; 98: 362-367). Br J Surg, 2011; 98866-7. | | PICO, study design |
| Silva AA,Camara CA,Martins A,Jr,Teles CJ,Terra JA, et al. Intraoperative cholangiography during elective laparoscopic cholecystectomy: selective or routine use? Acta Cir Bras, 2013; 28740-3. | | PICO, study design |
| Singh G,Gupta PC,Sridar G, Katariya RN. Role of selective intra-operative cholangiography during cholecystectomy. Aust N Z J Surg, 2000; 70106-9. | | PICO, study design |
| Slater K,Strong RW,Wall DR, Lynch SV. Iatrogenic bile duct injury: the scourge of laparoscopic cholecystectomy. ANZ J Surg, 2002; 7283-8. | | PICO, study design |
| Slim K, Martin G. Does routine intra-operative cholangiography reduce the risk of biliary injury during laparoscopic cholecystectomy? An evidence-based approach. J Visc Surg, 2013; 150321-4. | | Study design |
| Snow LL,Weinstein LS,Hannon JK, Lane DR. Evaluation of operative cholangiography in 2043 patients undergoing laparoscopic cholecystectomy: a case for the selective operative cholangiogram. Surg Endosc, 2001; 1514-20. | | Study design |
| Soper NJ, Brunt LM. The case for routine operative cholangiography during laparoscopic cholecystectomy. Surg Clin North Am, 1994; 74953-9. | | Study design |
| Soper NJ, Dunnegan DL. Routine versus selective intra-operative cholangiography during laparoscopic cholecystectomy. World J Surg, 1992; 161133-40. | | High risk of bias |
| Stuart SA,Simpson TI,Alvord LA, Williams MD. Routine intraoperative laparoscopic cholangiography. Am J Surg, 1998; 176632-7. | | Study design |
| Tabone LE,Sarker S,Fisichella PM,Conlon M,Fernando E,Yi S, et al. To 'gram or not'? Indications for intraoperative cholangiogram. Surgery, 2011; 150810-9. | | Study design |
| Taylor OM,Sedman PC,Jones BM,Royston CM,Arulampalam T, Wellwood J. Laparoscopic cholecystectomy without operative cholangiogram: 2038 cases over a 5-year period in two district general hospitals. Ann R Coll Surg Engl, 1997; 79376-80. | | Study design |
| Ueno K,Ajiki T,Sawa H,Matsumoto I,Fukumoto T, Ku Y. Role of intraoperative cholangiography in patients whose biliary tree was evaluated preoperatively by magnetic resonance cholangiopancreatography. World J Surg, 2012; 362661-5. | | PICO, study design |
| Van C,Prosmanne O,Gagner M,Pomp A,Deslandres E, Levesque HP. Routine operative cholangiography during laparoscopic cholecystectomy: feasibility and value in 107 patients. AJR Am J Roentgenol, 1993; 1601209-11. | | PICO, study design |
| Vecchio R,MacFadyen BV, Latteri S. Laparoscopic cholecystectomy: an analysis on 114,005 cases of United States series. Int Surg, 1998; 83215-9. | | Study design |
| Vezakis A,Davides D,Ammori BJ,Martin IG,Larvin M, McMahon MJ. Intraoperative cholangiography during laparoscopic cholecystectomy. Surg Endosc, 2000; 141118-22. | | PICO, study design |
| Videhult P,Sandblom G, Rasmussen IC. How reliable is intraoperative cholangiography as a method for detecting common bile duct stones? : A prospective population-based study on 1171 patients. Surg Endosc, 2009; 23304-12. | | Study design |
| Woods MS,Traverso LW,Kozarek RA,Donohue JH,Fletcher DR,Hunter JG, et al. Biliary tract complications of laparoscopic cholecystectomy are detected more frequently with routine intraoperative cholangiography. Surg Endosc, 1995; 91076-80. | | Study design |
| Yousefpour A,Kalbasi H,Setayesh A,Mousavi M,Hashemi A,Khodadoostan M, et al. Predictive value and main determinants of abnormal features of intraoperative cholangiography during cholecystectomy. Hepatobiliary Pancreat Dis Int, 2011; 10308-12. | | PICO, study design |
| Besselink MGH. Randomized clinical trial of routine on-table cholangiography during laparoscopic cholecystectomy. In: British journal of surgery; 2011. p 367. (fel artikel) | |  |
| Ding GQ, Cai W, Qin MF. Is intraoperative cholangiography necessary during laparoscopic cholecystectomy for cholelithiasis? World J Gastroenterol 2015;21:2147-51. | | High risk of bias |
| Enochsson L, Sandblom G, Österberg J, Thulin A, Hallerbäck BI, Persson G. Outcomes from the swedish registry of gallstone surgery and ERCP (GallRiks). Clinical consequences and implementation during a 10-year period. Gastroenterology 2015;148:S1160. | | Abstract |
| Grinberg R, Afthinos JN, Gibbs KE. National trend in intraoperative cholangiogram and common bile duct injury during cholecystectomy. Surgical Endoscopy and Other Interventional Techniques 2015;29:S534. | | Abstract |
| Hauer JM, Karesen R, Nygaard K. Consequences of routine peroperative cholangiography during cholecystectomy from gallstone disease: A prospective, randomized study. In: World-J-Surg; 1986. p 996-1002. | | Time period |
| Kumar A, Kumar U, Munghate A, Bawa A. Role of routine intraoperative cholangiography during laparoscopic cholecystectomy. Surg Endosc 2015;29:2837-40. | | Study design |
