# Supplementary material for: Routine versus selective intraoperative cholangiography during cholecystectomy: systematic review, meta-analysis and health economic model analysis of iatrogenic bile duct injury
Source: BJS Open. 2020 Dec 31;5(2):zraa032. doi: 10.1093/bjsopen/zraa032 (PMC7944855; doi:10.1093/bjsopen/zraa032)
Supplement: zraa032_Supplementary_Data [file zraa032_supplementary_data.zip › Supplement 3 Included studies.docx]

**Supplement 3** Intraoperative cholangiography or not in cholecystectomy - Included studies.

| **Author**  **Year**  **Reference**  **Country** | **Study period** | **N** | **Age span** | **Female/Male** | **Randomisa-tion** | **Indication for surgery** | **Primary endpoint**  **Bile duct injury (BDI)**  **Selective or no / routine IOC** | **Secondary endpoint** | **Harms** | **Follow-up time** | **Follow up, method** | **Risk of bias**  **Comments** |
| --- | --- | --- | --- | --- | --- | --- | --- | --- | --- | --- | --- | --- |
| Buddingh et al  2011  (33)  The Netherlands | 2004–2009 | 856 | ≥ 18 | F: 549 M: 307 | Cohort Before 2004-2006 (n=  421)/ after 2007–2009  (n=435) | All indications for gallbladder surgery  Selective 6% IOC  Routine 60% IOC | Major 8/0,  p 0.004  Minor 7/11, p 0.377 | Detection of CBD stones intraoperative 4/21 |  | ≥ 6 months | Review of patient records | Intermediate risk of bias  All major bile duct injuries  Routine IOC instituted 2007 and before that selective |
| Fletcher et al 1999  (34)  Australia | 1988–1994 | 19 176  1 683 OC,  7 493 LC  (1988–1990 7 113 OC  14 LC,  1991–1992 3 209 OC 2 526, LC  1993–1994 1 361 OC  4 953 LC61 + 4953) | <55: 10 941  55–64: 3 569  65-74: 3 112  >75: 1 524 | F: 13 967 M: 5 219 | – | All cholecystectomies | **BDI**  29/8 157, 0.55%/  15/11 029, 0.24%  **Bile leaks**  IOC 0.23%  no IOC 0.54%  **Injuries+bile leaks**  OR 0,50 (95% CI, 0.35; 0,70) p=0.0001 | – |  | 30 days | ≥ 2 days readmission | Intermediate risk of bias  “Major bile complications”  Shift in operative techniques during the study time, from open surgery only to predominantly laparoscopic |
| Flum et al  2001  (36)  USA | 1991–1998 | 30 639 LCs |  |  | Register cohort  Washington state |  | **Major BDI**  37/11 116 / 39/19 514  The rate of major injury in laparoscopic cholecystectomies performed without IOC was 3.3/1 000 compared with 2.0/1 000 in LCs with IOC  RR=1.7 (95% CI, 1.1; 2.6) |  |  |  |  | Intermediate risk of bias  All injuries major |
| Flum et al.  2003  (35)  USA, | 1992–1999 | 1 570 361 | – | – | Register cohort  Medicare |  | 5 531/956 655 (0.58%) / 2 380/ 613 706 (0.39%) p <0.001, RR=1.71 (95% CI, 1.38; 2.28) | – |  | 1 year | Search for ICD9 codes | Intermediate risk of bias  –surgical repair of CBD |
| Giger et al  2011  (37)  Switzerland, | 1995–2005 | 31 838 | Mean age 54.4 | Ratio  2:1 | – | All cholecystectomies | 61/20 196 (0.3%) / 40/11 642 (0.3%)  p=0.755  BDI detected postoperatively IOC 4/40 (10%)  No IOC 5/61 (8%)  p=0.737 | – | 2/101 with BDI died  41/31 737 without BDI died  OR 15.6 (95% CI, 3.7; 65.4) | Not stated– | – | Intermediate risk of bias  All types of BDI  “*Reasons for BDI were reported by the surgeons as follows: inadequate surgical exposure (12, 11,9 per cent), bleeding in Calot’s triangle (3, 3,0 per cent) and technical problems with the equipment (2, 2,0 per cent). In the remaining 84 patients (83,2 per cent), the surgeons were not aware of any conditions that complicated LE and/or promoted BDI.”* |
| Ragulin Coyne et al  2013  (38)  USA | 2004–2009 | Total: n=111 815  Selective: n=98 790  Routine: n=13 025 | 18–95 | **Male** Routine 33.1% Selective: 34% | Register Nationwide Inpatient Sample  Routine IOC surgeons (n=513), IOC rate of cholecystectomies=96%  Selective IOC surgeons (n=4 227), IOC rate of cholecystectomies=25% | LC | BDI  257/98 790, 0.26% / 33/13 025, 0.25%  Routine IOC provider vs selective  OR 0.96 (95% CI, 0.66; 1,41)  Multivariable |  | Routine IOC provider vs selective  In-hospital complications cardiovascular/myocardial infarction/deep venous thrombosis, gastrointestinal, pulmonary, urinary, infection  OR 1.10 (95% CI, 1.02; 1,20)  In-hospital mortality  OR 1.03 (95% CI, 0.76; 1,39) | - | - | Medium risk of bias  Only surgeons with 10 or more cholecystectomies per year  Type of BDI not specified.  “Common BDI” |
| Sheffield et al 2013  (39)  USA | 2001–2009 | 92 932 | > 66 (average 75) | F: 57 352 M: 35 580 | Retrospective cohort  Medicare + claims databases | Cholecystitis biliary colic or dyskinesia | BDI  No IOC 201/55 399, 0.36%  With IOC 79/37 533, 0.21%  OR 1.79 (95%CI, 1.35; 2.36) multilevel logistic regression  OR 1.26 (95%CI, 0.81; 1.96) after control for confounding with instrumental variable analysis | – | - | 1 year | Patient records | Medium risk of bias    “*Patients with CPT or ICD-9 procedure codes for choledochojejunostomy or hepaticojejunostomy within 1 year of surgery were considered to have had a major common duct injury during cholecystectomy”* |
| Waage et al 2006  (40)  Sweden | 1987–2001 | 152 776 | >15 | Ratio  2:1 | Register | All cholecystectomies, OC and LC | BDI  280/57 595 /  333/94 569  OR 0.66 (95% CI; 0.54; 0.79) multivariable  BDI omitting patients with CBD stones IOC/no IOC  OR 0.75 (95% CI; 0.59; 0.92) multivariable | - |  | 1 year |  | Medium risk of bias  “*To define BDI cases, we first selected patients in this population who during the index procedure or within 1 year after it had also undergone any reconstructive biliary procedures*  *registered using ICD-9 and ICD-10 procedure codes”*  *“There was a small to moderate long-term increase*  *in the risk of BDI after the introduction of laparoscopic cholecystectomy compared with the prelaparo-scopic era.”* |

Reference numbers refers to the main document

BDI = Bile duct injury; CBD = Common bile duct; IOC= Intraoperative cholangiography; LC = Laparoscopic cholecystectomy

**References**

33. Buddingh KT, Weersma RK, Savenije RA, van D, G M, Nieuwenhuijs VB. Lower rate of major bile duct injury and increased intraoperative management of common bile duct stones after implementation of routine intraoperative cholangiography. J Am Coll Surg. 2011;213:267-74.

34. Fletcher DR, Hobbs MS, Tan P, Valinsky LJ, Hockey RL, Pikora TJ, et al. Complications of cholecystectomy: risks of the laparoscopic approach and protective effects of operative cholangiography: a population-based study. Ann Surg. 1999;229:449-57.

35. Flum DR, Dellinger EP, Cheadle A, Chan L, Koepsell T. Intraoperative cholangiography and risk of common bile duct injury during cholecystectomy. JAMA. 2003;289:1639-44.

36. Flum DR, Koepsell T, Heagerty P, Sinanan M, Dellinger EP. Common bile duct injury during laparoscopic cholecystectomy and the use of intraoperative cholangiography: adverse outcome or preventable error? Arch Surg. 2001;136:1287-92.

37. Giger U, Ouaissi M, Schmitz SF, Krahenbuhl S, Krahenbuhl L. Bile duct injury and use of cholangiography during laparoscopic cholecystectomy. Br J Surg. 2011;98:391-6.

38. Ragulin-Coyne E, Witkowski ER, Chau Z, Ng SC, Santry HP, Callery MP, et al. Is routine intraoperative cholangiogram necessary in the twenty-first century? A national view. J Gastrointest Surg. 2013;17:434-42.

39. Sheffield KM, Riall TS, Han Y, Kuo YF, Townsend CM, Jr, et al. Association between cholecystectomy with vs without intraoperative cholangiography and risk of common duct injury. JAMA. 2013;310:812-20.

40. Waage A, Nilsson M. Iatrogenic bile duct injury: a population-based study of 152 776 cholecystectomies in the Swedish Inpatient Registry. Arch Surg. 2006;141:1207-13.
